# Incidence and factors influencing neck shortening after screw fixation of femoral neck fractures with the femoral neck system

**DOI:** 10.1186/s13018-023-03787-5

**Published:** 2023-04-24

**Authors:** Kai Wang, Dongze Lin, Peisheng Chen, Chaohui Lin, Tianxuan Feng, Jiajie Liu, Shunze Zheng, Yaqian Liang, Jirui Ouyang, Yubo Cui, Fengfei Lin

**Affiliations:** 1 Department of Orthopaedics, Fujian Provincial Clinical Medical Research Center for First Aid and Rehabilitation in Orthopaedic Trauma, Fuzhou Second Hospital of Xiamen University, The Third Clinical Medical College of Fujian Medical University, Fuzhou, 350007 China; 2 The Second Clinical Medical College of Fujian University of Traditional Chinese Medicine, Fuzhou, Fujian China

**Keywords:** Femoral neck fracture, Fracture shortening, Internal fixation, Incidence, Comminution

## Abstract

**Objective:**

To investigate the effects of postoperative femoral neck shortening in patients with femoral neck fractures fixed with femoral neck system screws (FNS) and to explore the factors influencing femoral neck shortening.

**Method:**

To retrospectively analyze the data of 113 patients with femoral neck fractures admitted to the Second Hospital of Fuzhou City, affiliated with Xiamen University, between December 2019 and January 2022. Of these, 87 patients were followed up for more than 12 months, 49 men and 38 women: 36 cases of Garden I and II fractures and 51 cases of Garden III and IV fractures, to record the patient's hip Harris score at 12 months postoperatively. Patients were divided into femoral neck shortening group and femoral neck no shortening group according to their regular postoperative follow-up radiographic measurements. To count the incidence of femoral neck shortening, a comparison of postoperative complication rates and hip Harris scores between the two groups was made. Statistical comparison of the two groups and a multifactorial logistic regression analysis were also performed to analyze the factors affecting femoral neck shortening.

**Results:**

All 87 patients were followed up for more than 12 months after surgery. In 34 of these cases, neck shortening occurred, and the incidence rate was 39.1%. 15 cases of severe shortening, incidence of 17.2%; fracture healing 84 cases, fracture healing rate of 96.5%. The hip Harris score was 83.99 (81.95, 89.20) in the neck shortening group at 12 months postoperatively, 90.87 (87.95, 94.80) for the group without neck shortening; the difference between the two groups was statistically significant (*P* < 0.01). 32 cases of fracture healing in the neck shortening group at 12 months after surgery, fracture healing rate of 94.1%; 52 cases healed without neck shortening group, fracture healing rate of 98.1%. The difference between the two groups was not statistically significant (*P* = 0.337). High incidence of neck shortening after FNS fixation of femoral neck fractures, cortical comminution of the severed end, fracture fractionation and quality of reduction were significantly correlated with neck shortening.

**Conclusion:**

High incidence of postoperative neck shortening after internal fixation of femoral neck fractures with the femoral neck system, the cortical comminution, the type of fracture, and the quality of fracture reduction are the influencing factors; femoral neck shortening can affect postoperative hip function, but does not affect fracture healing.

**Supplementary Information:**

The online version contains supplementary material available at 10.1186/s13018-023-03787-5.

## Introduction

Fracture of the neck of the femur is a devastating injury, according to relevant information estimates, Worldwide, the number of new hip fractures will increase by approximately 6.3 million by mid-century [1, 2], this is a heavy burden for the health care system and economic and social development. The incidence of osteonecrosis and femoral head necrosis after internal fixation of femoral neck fractures is still as high as 20–30%, respectively [3, 4], with unsatisfactory treatment results, which are also called “unresolved fractures.”

Current research on femoral neck fractures has traditionally focused on fracture healing rates, surgical revision rates, and mortality, and little is known about the physical limitations of the patient's body due to internal fixation and the recovery of postoperative function. Internal fixation treatment modalities include multiple parallel screws, power hip screws, and power cross-nailing of the femoral neck. This type of internal fixation allows the fracture end to be axially loaded postoperatively and promotes fracture healing by compressing the fracture end to a certain extent. However, this mechanism may lead to femoral neck shortening, and the importance of the complete force arm of the hip abductor muscle group has been demonstrated in hip replacement [5, 6], and shortening of the force arm of the abductor muscle group can lead to detrimental effects on hip mobility, with studies indicating that postoperative femoral neck shortening can reduce walking speed and lead to permanent physical limitations [7]. In a retrospective study conducted by Stockton DJ, the incidence of femoral neck shortening after femoral neck fracture in young adults was noted to be as high as 54% [8]; however, in the field of trauma, there seems to be more focus on osteonecrosis and femoral head necrosis, and the adverse effects of femoral neck shortening are less reported.

In contrast, a new biomechanical study recently showed that the femoral neck system (FNS) has greater resistance to rotation and shear and better biomechanical properties, but is deficient in femoral neck shortening [9]. The incidence of femoral neck shortening after FNS for femoral neck fractures has been reported clinically differently, and the purpose of our present study was to investigate the incidence and shortening distance of its postoperative femoral neck shortening and to analyze the factors influencing neck shortening after internal fixation.

## Materials and methods

Case inclusion and exclusion criteria.

Case inclusion criteria: (1) unilateral femoral neck fracture seen within 2 weeks after the injury; (2) treated with FNS internal fixation; (3) complete follow-up data ≥ 12 months; and (4) normal hip movement on the affected side before the injury.

Case exclusion criteria: (1) pathological fractures or old fractures; (2) acetabular dysplasia; (3) long-term hormone use; (4) previous cardiovascular and cerebrovascular diseases; and (5) incomplete clinical follow-up data.

### General information

In this study, 113 patients with fresh femoral neck fractures met the inclusion criteria, of which 25 did not complete the follow-up and 1 had a re-fracture due to a postoperative fall, for a total of 87 patients who completed more than 12 months of follow-up. There were 49 male cases and 38 female cases; 46 left-sided cases and 41 right-sided cases. The fractures were classified by Garden and Pauwels: 23 cases of Garden I, 13 cases of Garden II, 12 cases of Garden III, and 39 cases of Garden IV; Pauwels I: 8 cases; Pauwels II: 30 cases; and Pauwels III: 49 cases.

For imaging data, 2 professionally trained orthopedic surgeons measured the imaging data separately, and any disputes were resolved by discussion and negotiation; the patient's radiographs were used to assess fracture staging (Garden Typing, Pauwels Typing).

This study complied with the Declaration of Helsinki as revised in 2013 and was approved by the Medical Ethics Committee of Fuzhou Second Hospital, Xiamen University. All patients gave informed consent and signed the informed consent form.

### Surgery method

All patients were placed under general anesthesia with tracheal intubation in the supine “lithotomy” position with sterile towels. At the proximal end of the femur, 2 kerf pins are prepositioned laterally along the axis of the femoral neck, not exceeding the fracture line, according to the preoperative plan, a threaded bone traction nail can be inserted approximately 2–3 cm below the lateral apex of the greater trochanter for anterior and posterior positions that require correction of displacement, combined with longitudinal traction of the lower extremity, in the presence of femoral rotation, 2 kerf pins were placed in the femoral head to reset the femoral head rotation. After repositioning, anterior–posterior and table-piercing radiographs were taken using the intraoperative C-arm, and the position met the repositioning requirements and was fixed by inserting two preplaced kerf pins. A guide pin was inserted under fluoroscopy, parallel to the axis of the femoral neck, and adjusted so that it was located approximately 10% inferior to the mid-axis of the femoral neck in the anterior–posterior position and on the mid-axis of the femoral neck in the lateral position, and the corresponding length of FNS was selected for fixation after measurement, and the wound was irrigated and sutured.

### Postoperative treatment and observation index

Cefazolin sodium was given within 24 h after surgery to prevent infection, and low-molecular heparin calcium was injected on the first day after surgery to prevent deep vein thrombosis. Patients were instructed to perform foot flexion and extension exercises in a non-weight-bearing position, and all patients were kept in a non-weight-bearing position for at least 4 weeks after surgery. In patients with progressive growth of bone scabs on imaging observed at follow-up, patients were instructed to be partially weight-bearing, weighing approximately 20–30 kg, with the patient's weight-bearing status increasing at what rate determined by 2 physicians, with regular postoperative outpatient review at 1, 2, 3, 6, and 12 months.

The hip Harris score was used to assess the functional outcome of the affected limb during the follow-up of all patients included in the study. It includes four components: pain level, functional status, deformity, and range of motion. The total Harris score is 100, and the better the recovery of the affected limb after surgery, the higher the score.

### Main observation indicators and measurement methods

These include non-healing fractures, femoral head necrosis, femoral neck shortening, and failure of internal fixation. Fracture healing is defined as a bone scab bridging the fracture end or a bone trabecula across the fracture end within 6 months, and vice versa is considered non-healing [10]. Femoral neck shortening was defined as shortening of ≥ 5 mm on final follow-up radiographs compared with the immediate postoperative period of the fracture [11, 12]. Femoral head necrosis: assessment based on Ficat and Arlet's radiological criteria [13]. Internal fixation device disorders include main nail cut out, fracture, screw loosening, etc.

Definition of femoral neck shortening: Femoral neck shortening 0–5 mm was defined as no shortening; 5–10 mm as mild shortening; and > 10 mm as severe shortening [14].

Measurement of femoral neck shortening: Both the femoral neck power cross-nailing system and the sliding hip screw are angled fixation devices that achieve fracture healing by a sliding compression mechanism. The difference between the sliding space compression distance on immediate postoperative radiographs and the sliding space compression distance on postoperative follow-up radiographs is based on the femoral neck shortening length of the pacs real-time system at the same magnification.

The quality of fracture repositioning in the patients of this study was classified according to the Garden alignment index [15]. The angle between the femoral head pressure trabeculae and the medial cortex of the femoral stem is 160° in normal hip orthopantomogram and 180° between the medial axis of the femoral head and the medial axis of the femoral neck in lateral radiograph. Based on the Garden Reset Index, the reset quality is classified into four levels.

**Table:** 

	Garden index
Level I	Anteroposterior 160°, Lateral 180°
Level II	Anteroposterior 155°–160°, Lateral 180°
Level III	Anteroposterior 150°–155°, Lateral > 180°
Level IV	Anteroposterior < 150°, Lateral > 180°

### Statistical methods

Statistical analysis was performed by applying IBM.SPSS Statistics 26 statistical software. For the measurement data, the Shapiro–Wilk test was used to determine whether the data conformed to a normal distribution, where age and body mass index conformed to a normal distribution and the variance was chi-square, expressed as *x* ± *s*. Two independent samples t test was used for comparison between the two groups of patients. Harris score and femoral neck shortening length did not conform to a normal distribution and were expressed as M (P_25_, P_75_), and the Mann–Whitney *U* test was used to compare between the two groups of patients. Count data such as gender, mechanism of injury, smoking and alcohol history, laterality, fracture Garden's staging, Pauwels’ staging, quality of immediate postoperative reduction, reduction and complication rate were compared between the two groups using the *χ*^2^ test. Pauwels typing and early complication rates were compared using Fisher's exact probability method. *P* < 0.05 was considered statistically significant. Whether the broken end cortex was comminuted, the type of fracture and the quality of immediate postoperative reduction were used as independent variables, and whether neck shortening occurred postoperatively was used as the dependent variable, which was first analyzed univariately using the *χ*^2^ test, and then, the independent variables with statistically significant differences confirmed by univariate analysis were brought into a dichotomous logistic regression model for multivariate analysis, and differences were considered statistically significant at *P* < 0.05. The images were created using GraphPad Prism 9 software.

## Results

### General results

There was no statistically significant difference between the two groups in the comparison of general preoperative data such as age, sex, side, history of smoking and alcohol, and mechanism of injury (*P* > 0.05), while there was a statistically significant difference between the two groups in the comparison of fracture Garden typing, Pauwels typing, cortical comminution, and quality of reduction (*P* < 0.05).

### Incidence of neck shortening, fracture healing rate, shortening distance and hip function score

Among the 87 patients with femoral neck fractures, 34 cases had neck shortening, with an incidence of 39.1%, and 15 cases had severe shortening, with an incidence of 17.2%; 84 cases had fracture healing, with a fracture healing rate of 96.5%. The hip Harris score at 12 months postoperatively was 83.99 (81.95, 89.20) in the neck shortening group and 90.87 (87.95, 94.80) in the group without neck shortening, which was statistically significant compared with the two groups (Table 1). There was no statistical difference between the two groups in terms of osseous nonunion, femoral head collapse necrosis, and failure of internal fixation (Table 2).

**Table 1:** Comparison of general information of neck shortening group and no neck shortening group

Patient characteristics	Neck shortening group (*n* = 34)	Neck without shortening group (*n* = 53)	*P*
Age (years)	49.04 ± 9.430	47.32 ± 11.514	0.158
Sex (cases)			0.149
Female	12 (35.3%)	26 (49.1%)	
Male	22 (64.7%)	27 (50.9%)	
Side			0.138
Left	15 (44.1%)	31 (58.5%)	
Right	19 (55.9%)	22 (41.5%)	
BMI	22.97 ± 2.696	22.89 ± 2.515	0.885
Smoke and drink			0.436
Yes	3 (8.8%)	3 (5.7%)	
No	31 (91.2%)	50 (94.3%)	
Injury mechanism			0.228
High-energy	19 (55.9%)	24 (45.3%)	
Low-energy	15 (44.1%)	29 (54.7%)	
Garden type			< 0.001
I, II	3 (8.8%)	33 (62.2%)	
III, IV	31 (91.2%)	20 (37.7%)	
Pauwels classification			0.019
I	3 (8.8%)	5 (9.4%)	
II	6 (17.6%%)	24 (45.3%)	
III	25 (73.5%)	24 (45.3%)	
Cortical crushing			< 0.001
Yes	28 (82.4%)	15 (28.3%)	
No	4 (9.5%)	38 (71.7%)	
Quality of fracture repositioning			0.001
Level I, II reset	17 (50.0%)	51 (96.2%)	
Level III, IV reset	17 (50.0%)	2 (3.8%)	
Harris rating	83.99 (81.95,89.20)	90.87 (87.95,94.80)	0.001
Shortening distance	9.70 (7.19, 11.73)	2.66 (1.32,3.89)	< 0.001

**Table 2:** Comparison of postoperative complications between the neck shortening group and the group without neck shortening

	Neck shortening group (*n* = 34)	Neck without shortening group (*n* = 53)	*P*
Osteointegration	3 (8.8%)	0 (1.9%)	0.056
Femoral head necrosis	1 (2.9%)	0	0.391
Failure of internal fixation	3 (8.8%)	0	0.056

### Factors influencing femoral neck shortening

Multifactorial logistic regression analysis showed that cortical comminution of the severed end, fracture staging (Garden type III and IV), and postoperative quality of reduction (grade III and IV) were significantly associated with neck shortening (all *P* < 0.05). See Table 3.

**Table 3:** Multi-factor logistic regression analysis of postoperative neck shortening after femoral neck fracture

Influencing factors	B	Wald	OR	*P*	95%CI
Cortical comminution	1.776	5.875	5.903	0.015	(1.405,24.831)
Garden typing (III, IV)	1.723	4.656	5.604	0.031	(1.171,26.815)
Pauwels typing (III)	− 0.439	0.707	0.645	0.535	(0.161,2.577)
Reset quality (III, IV)	1.701	3.886	5.478	0.049	(1.010,29.719)

### Comparison of femoral neck shortening between the two groups at postoperative follow-up


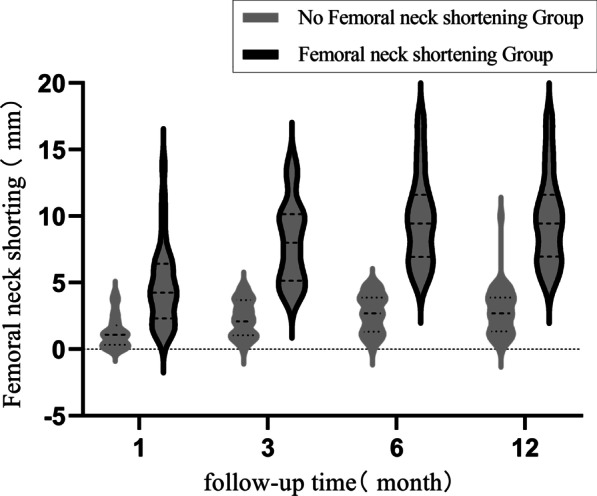


## Discussion

Femoral neck fracture is a common type of fracture in clinical practice, and its incidence is increasing year by year as the aging society intensifies. There is a consensus that the preferred treatment for young patients with femoral neck fractures is internal fixation surgery, which strives for anatomic repositioning to bring the patient to the pre-injury level as early as possible. However, there are still some noteworthy problems in the treatment, such as a significant proportion of patients in the postoperative follow-up process showed femoral neck shortening on imaging and gait manifested as claudication, the frequency of this phenomenon, the impact on the patient's hip function and the influencing factors are less attention in the current relevant studies.

### Incidence of neck shortening

**Fig. 1:**
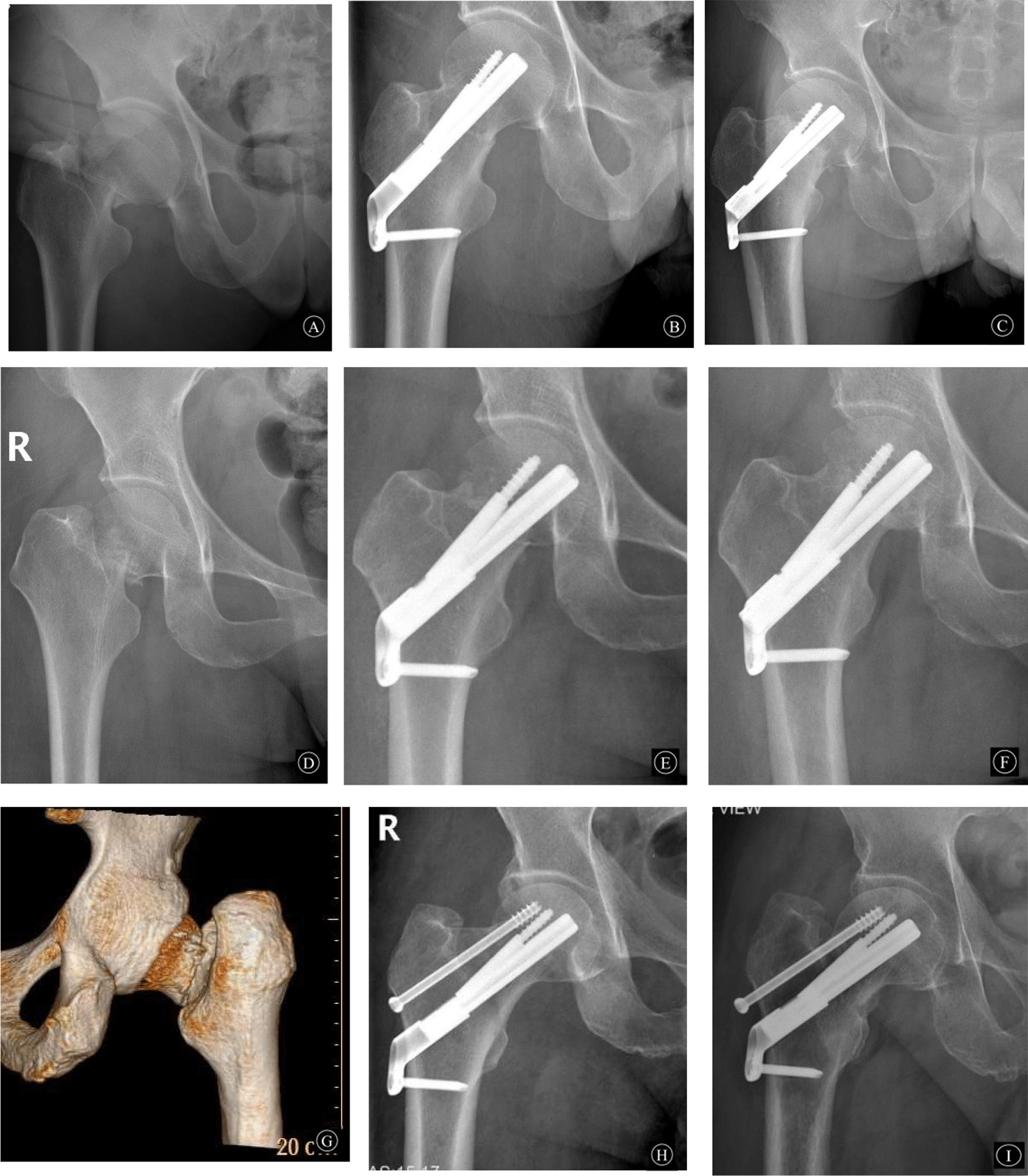
**A**, **B**, **C** are 53-year-old men, **A** preoperative hip orthogonal position shows right femoral neck fracture (Garden type IV), **B** postoperative hip orthogonal position FNS sliding space is not limited (20 mm); **C** 6 months postoperative femoral neck is severely shortened, FNS still has about 5 mm sliding space. **D**, **E**, **F** are 58-year-old women, **D** preoperative hip orthogonal position shows right femoral neck fracture (Garden type IV), **E** postoperative hip orthogonal position FNS sliding space restricts sliding space (about 5 mm), **F** 6 months postoperative femoral neck mild shortening (shortening < 5 mm). **G**, **H**, and **I** were 54-year-old men; **G**'s preoperative CT 3D reconstruction of the hip showed posterior medial cortical comminution; **H**'s postoperative hip was in an orthogonal position near anatomic reset; **I**'s right hip was in an orthogonal position 6 months postoperatively with severe shortening of the femoral neck (< 10 mm of shortening)

The incidence of femoral neck shortening after femoral neck fixation with the femoral neck system (FNS) is poorly reported in the literature; in a case study of FNS for femoral neck fractures, the incidence of neck shortening was 28% [16], whereas in the current study, the incidence was 39.1%, and this may be related to the large proportion of Garden III and IV types in the current study. During the follow-up of this study, we found that the time period of postoperative shortening was concentrated at 3 months postoperatively (Fig. 1), and persistent shortening rarely occurred after 3 months. This on one hand may be related to the presence of cortical comminution, although the length of the femoral neck can be restored after intraoperative traction and repositioning, but because the FNS is an angular fixation device, it cannot be fixed to the comminuted cortex, and the fracture end will undergo continuous micromovement, combined with the mechanism of sliding compression healing, the FNS reserves 20 mm sliding compression space (Fig. 1A–C), and the resistance to restrict shortening comes from the cortical counteraction. Poor repositioning, bone defect, cortical comminution, etc., lead to cortical failure to form a buttress, which is prone to excessive postoperative shortening and no length-maintaining fixation of the femoral neck, and limiting the sliding space may reduce the distance of femoral neck shortening (Fig. 1D–F). On the other hand, it may be related to local bone resorption during the healing process of the fracture end after surgery.

### Risk factors for femoral neck shortening

The current literature focuses on the fields of osteonecrosis and femoral head necrosis, and less attention has been paid to femoral neck shortening and the factors affecting it. In this study, several variables were selected to explore their correlation with femoral neck shortening, and through binary logistic regression analysis, it was found that femoral neck shortening was most associated with cortical comminution, fracture type, and quality of postoperative repositioning.

Femoral neck fractures are often associated with cortical comminution, especially the posterior medial cortex, with an incidence of up to 90% [17], and more severe bone resorption after repositioning, with continued compression of the comminuted cortex under compression of the longitudinal load along the internal fixation after weight-bearing of the hip joint begins, leading to more severe femoral neck shortening [18]. Current studies have reported that in femoral neck fractures with posterior medial cortical comminution, placement of a single screw can compensate for the negative effects of cortical loss [19, 20], in the current study, there were unincluded patients with this type of fixation and postoperative femoral neck shortening < 10 mm (Fig. 1G–I).

Currently, Garden and Pauwels typing is most commonly used in femoral neck fractures; Garden typing is classified according to the degree of fracture displacement; Garden I and II are stable fractures; Garden III and IV are displaced fractures. Garden III and IV fractures with large initial fracture displacement and severe bone destruction are considered to be factors that affect the stability and healing of femoral neck fractures. Stockton DJ et al. performed a retrospective study analyzing the imaging data of 65 patients and showed that displaced fractures were on average 5.9 mm more shortened than undisplaced fractures [8]. Displaced fractures were also noted as an independent predictor of femoral neck shortening in a prospective multicenter trial published by Gerard P. Slobogean et al. in 2017 [21]. In addition, it has also been noted in a large body of the literature that cortical comminution and displaced fractures correlate with femoral neck shortening [7, 14, 22]. Pauwels typing is based on the angle between the distal fracture line and the horizontal line (Pauwels angle), type I: < 30°; type II: 30–50°; type III: > 50°. Pauwels typing and femoral neck shortening did not constitute independent risk factors in the present study, probably related to the predominance of Pauwels type III in the present study, with large Pauwels angles and a fracture end dominated by shear forces and pressure loads not acting well to pressurize the fracture end.

Femoral neck fractures are treated by striving for anatomical repositioning and restoring the concentric matching of the femoral head to the acetabulum. In 1971 Garden et al. performed a minimum of 3 years of clinical follow-up after internal fixation treatment of more than 400 femoral neck fractures and found that there was a graded relationship between the quality of reduction of femoral neck fractures and postoperative complications; as the quality of reduction decreased, the incidence of postoperative complications increased, and proposed the Garden reduction alignment index, which was divided into four grades and is used today [15]. Yoram A. Weil et al. found in a study of 41 cases of femoral neck fractures treated with three hollow nails that the incidence of postoperative neck shortening could be as high as 51% with up to 75% non-anatomic reduction [23]. This suggests that when the quality of the repositioning is unsatisfactory, partial insertion of the fracture end aggravates shortening when subjected to human loading, and even necrosis of the femoral head can occur under long-term stress stimulation [12, 24]. Since collapsed femoral head necrosis usually does not show clinical symptoms until 1 year, or even longer, after surgery, and most patients have a low detection rate in the early stage of the lesion, there are also some patients who may be missed or misdiagnosed, etc. This also means that the signs of femoral head necrosis are not yet fully detected based only on the 12-month postoperative follow-up of patients, which is the main reason why the incidence of femoral head necrosis in this study is lower than the results reported in the literature. Therefore, we still need to follow the patients for a long time and continue to observe the occurrence of postoperative femoral head necrosis.

### Effect of femoral neck shortening on fracture healing and joint function

The effect of postoperative neck shortening on fracture healing and postoperative functional recovery after FNS fixation of femoral neck fractures is one of the current hot issues of concern. The current study showed a healing rate of (32/34) 94.1% in the neck shortening group and (52/53) 98.1% in the group without neck shortening, with no statistically significant difference between the two groups (*P* > 0.05). Although neck shortening has no significant effect on fracture healing, it can cause a decrease in hip function. In an analysis of a prospective multicenter study on the prognosis of hip fractures in China published by Slobogean et al. in 2017, it was noted that femoral neck shortening ≥ 1 cm was associated with a poorer functional prognosis after surgery in young patients with femoral neck fractures [21]. A multicenter study of 660 patients by Zlowodzki showed a gradient decrease in SF-36 scores in the no-shortening group (< 5 mm), moderate shortening group (5–10 mm), and moderate shortening group (≥ 10 mm) and that neck shortening was the only predictor of physical function scores [12]. In this study, we used the hip Harris score as an index to evaluate the recovery of hip function, and at the 12-month postoperative follow-up, the score was 83.99 (81.95, 89.20) in the neck shortening group and 90.87 (87.95, 94.80) in the neck non-shortening group, and the difference between the two groups was statistically significant, and the gap on the Harris score was mainly in the use of aids, abnormal gait, etc. The reason for this analysis may be the shortening of the force arm and increased load on the hip abductor muscle group due to neck shortening, thus failing to balance the vertical stress from the pelvis and causing varying degrees of claudication. In addition, the lateral plate placed on the lateral side of the femur may cause irritation to the surrounding soft tissues, thus causing discomfort to the patient and affecting the functional activity of the hip joint.

In conclusion, the rate of femoral neck shortening after FNS internal fixation is high, and femoral neck shortening affects the hip function of patients, and the cortical comminution of the broken end, the type of fracture, and the quality of fracture reduction are the influencing factors of femoral neck shortening. However, the limitations of this study are the radiographic set affected by position and the individual incomplete follow-ups, which may be altered by radiographic lower limb position despite efforts to standardize the measurement of femoral neck shortening values; in addition, femoral neck shortening was measured after repositioning and fixation, and for patients with shortening after repositioning, subsequent measurements may be more distant compared to the true shortening. The measurement of femoral neck shortening in this study was measured by two researchers to group the measurements, and any inconsistencies were coordinated and resolved. In addition, to reduce bias errors, data measurement was not performed by the same personnel as the hip Harris score (Additional file 1).

## Supplementary Information

**Additional file 1.** Data content of this study.

## Data Availability

All data analyzed during this study are included in this published article.

## References

[1] Zuckerman JD (1996). Hip fracture. N Engl J Med.

[2] Raaymakers EL (2006). Fractures of the femoral neck: a review and personal statement. Acta Chir Orthop Traumatol Cech.

[3] Mahmoud SS, Pearse EO, Smith TO (2016). Outcomes of total hip arthroplasty, as a salvage procedure, following failed internal fixation of intracapsular fractures of the femoral neck: a systematic review and meta-analysis. Bone Joint J.

[4] Haidukewych GJ, Rothwell WS, Jacofsky DJ (2004). Operative treatment of femoral neck fractures in patients between the ages of fifteen and fifty years. J Bone Joint Surg Am.

[5] Mcgrory BJ, Morrey BF, Cahalan TD (1995). Effect of femoral offset on range of motion and abductor muscle strength after total hip arthroplasty. J Bone Joint Surg Br.

[6] Charles MN, Bourne RB, Davey JR (2005). Soft-tissue balancing of the hip: the role of femoral offset restoration. Instr Course Lect.

[7] Zielinski SM, Keijsers NL, Praet SF (2013). Femoral neck shortening after internal fixation of a femoral neck fracture. Orthopedics.

[8] Stockton DJ, Lefaivre KA, Deakin DE (2015). Incidence, magnitude, and predictors of shortening in young femoral neck fractures. J Orthop Trauma.

[9] Stoffel K, Zderic I, Gras F (2017). Biomechanical evaluation of the femoral neck system in unstable Pauwels III femoral neck fractures: a comparison with the dynamic hip screw and cannulated screws. J Orthop Trauma.

[10] Butt MF, Dhar SA, Gani NU (2008). Delayed fixation of displaced femoral neck fractures in younger adults. Injury.

[11] Zlowodzki M, Ayeni O, Petrisor BA (2008). Femoral neck shortening after fracture fixation with multiple cancellous screws: incidence and effect on function. J Trauma.

[12] Zlowodzki M, Brink O, Switzer J (2008). The effect of shortening and varus collapse of the femoral neck on function after fixation of intracapsular fracture of the hip: a multi-centre cohort study. J Bone Joint Surg Br.

[13] Ficat P, Arlet J (1973). Pre-radiologic stage of femur head osteonecrosis: diagnostic and therapeutic possibilities. Rev Chir Orthop Reparatrice Appar Mot.

[14] Felton J, Slobogean GP, Jackson SS (2019). Femoral neck shortening after hip fracture fixation is associated with inferior hip function: results from the FAITH trial. J Orthop Trauma.

[15] Garden RS (1974). Reduction and fixation of subcapital fractures of the femur. Orthop Clin North Am.

[16] Tang Y, Zhang Z, Wang L (2021). Femoral neck system versus inverted cannulated cancellous screw for the treatment of femoral neck fractures in adults: a preliminary comparative study. J Orthop Surg Res.

[17] Collinge CA, Mir H, Reddix R (2014). Fracture morphology of high shear angle "vertical" femoral neck fractures in young adult patients. J Orthop Trauma.

[18] Liu Y, Ai ZS, Shao J (2013). Femoral neck shortening after internal fixation. Acta Orthop Traumatol Turc.

[19] Kauffman JI, Simon JA, Kummer FJ (1999). Internal fixation of femoral neck fractures with posterior comminution: a biomechanical study. J Orthop Trauma.

[20] Rajnish RK, Haq RU, Aggarwal AN (2019). Four screws diamond configuration fixation for displaced, comminuted intracapsular fracture neck femur in young adults. Indian J Orthop.

[21] Slobogean GP, Stockton DJ, Zeng BF (2017). Femoral neck shortening in adult patients under the age of 55 years is associated with worse functional outcomes: analysis of the prospective multi-center study of hip fracture outcomes in China (SHOC). Injury.

[22] Huang TW, Hsu WH, Peng KT (2011). Effect of integrity of the posterior cortex in displaced femoral neck fractures on outcome after surgical fixation in young adults. Injury.

[23] Weil YA, Khoury A, Zuaiter I (2012). Femoral neck shortening and varus collapse after navigated fixation of intracapsular femoral neck fractures. J Orthop Trauma.

[24] Nanty L, Canovas F, Rodriguez T (2019). Femoral neck shortening after internal fixation of Garden I fractures increases the risk of femoral head collapse. Orthop Traumatol Surg Res.

